# Warfare: Iraq’s Toxic Shipwrecks

**DOI:** 10.1289/ehp.113-a230

**Published:** 2005-04

**Authors:** Valerie J. Brown

Iraq’s coastline consists of a 36-mile stretch along the north end of the Persian Gulf. The country has only two deep-water ports, Umm Qasr and Az Zubayr. Three wars—the Iran–Iraq War from 1980 to 1988, the 1991 Gulf War, and the U.S. invasion of Iraq in 2003—have cluttered northern gulf waters with a welter of sunken ships, many of which still hold petroleum products, unexploded ordnance, and possibly rocket fuel, propellants, and toxic chemicals. Many of the ships are leaking. Little is known about the environmental health consequences of these marine obstacles and their contents, but a recent report by the United Nations Development Programme (UNDP) looks at the potential environmental hazards posed by the sunken ships.

In preparing *Iraqi Waterway Project Wreck Removal: Environmental Damage Limitation Survey*, published in October 2004, a UNDP team assisted by International Atomic Energy Agency marine experts and two French water pollution agencies inspected 40 wrecks, identified 12 more by sonar, and collected 198 sediment samples for analysis. The team estimates that more than 260 sunken ships—including tankers, tugs, barges, and patrol boats—clog the local waters. “Virtually all of these vessels are slowly leaking substances that are damaging to marine life and people alike,” states the report. “Even if the vessel was not carrying a hazardous cargo, the engine room will typically contain substances such as fuel oil, lubricating oil, battery acid, hydraulic fluid, and asbestos.”

Silting is a major problem in Iraqi ports, as with most harbors. But dredging cannot proceed safely or commerce resume fully until more of the wrecks have been cleared away—a process made complex and dangerous by the possibility of ordnance detonation and the turbidity produced by the strong gulf current. The current flows counterclockwise toward Kuwait, and it may carry pollution toward Kuwaiti desalination plants along the Persian Gulf coast. Approximately 70–90% of the people in the gulf region get their fresh water from desalination plants, according to *The Economic and Environmental Impact of the Gulf War on Kuwait and the Persian Gulf*. This report appears in the Trade and the Environment Database, a project of American University in Washington, D.C.

The UNDP found that oil is the worst problem related to the sunken ships, stating that “significant oil pollution was painfully evident even without any sample analysis.” Much of the oil is crude, bunker, and diesel grades. Such oils contain many hydrocarbon compounds, including benzene, propane, acetylene, naphtha, and kerosene, all of which can cause health effects. Benzene, for example, can cause dizziness, tremors, anemia, and leukemia, according to the Agency for Toxic Substances and Disease Registry (ATSDR), and is classified as a known carcinogen by the National Toxicology Program. Depending on the exposure pathway, fuel oils can cause nausea, loss of appetite, poor coordination, kidney damage, heightened blood pressure, and other problems, according to the ATSDR.

Heavy metals were generally found in relatively small quantities, although one sediment sample from inside a wreck did contain elevated lead levels. Radioactivity was consistent with natural distribution of uranium in the Earth’s crust. The survey found low concentrations of polycyclic aromatic hydrocarbons and no evidence of organochlorines such as polychlorinated biphenyls or DDT. The report also cites a 1994 UK government letter to the Security Council expressing concern that Iraqi patrol boats and hovercraft sunk in the first Gulf War may have carried chemical weapons canisters which could begin to leak.

The UNDP survey was part of a series of projects to clean up Iraqi waters and bring the ports to full capacity. Before the report was issued, 31 wrecks had already been removed, according to Michel Gautier, UNDP–Iraq infrastructure manager. The UNDP proposes to continue wreck removal activities, and collection and treatment of the remaining oil in the wrecks in both Iraqi and adjoining Kuwaiti waters, with the focus on identifying and removing the most obstructive and dangerous wrecks.

The Persian Gulf isn’t the only place threatened by toxic wrecks. A 22 January 2005 article in *New Scientist* reported that the U.S. liberty ship *Richard Montgomery*, scuttled in the Thames during World War II, might release and detonate its payload of TNT in the next 20 years. And a 2001 cyclone plastered the Micronesian atoll of Ulithi with oil and gasoline from the U.S.S. *Mississinewa*, sunk during World War II and estimated to still contain 36 million liters of oil, according to the Vanuatu news organization Port Vila Presse.

## Figures and Tables

**Figure:**
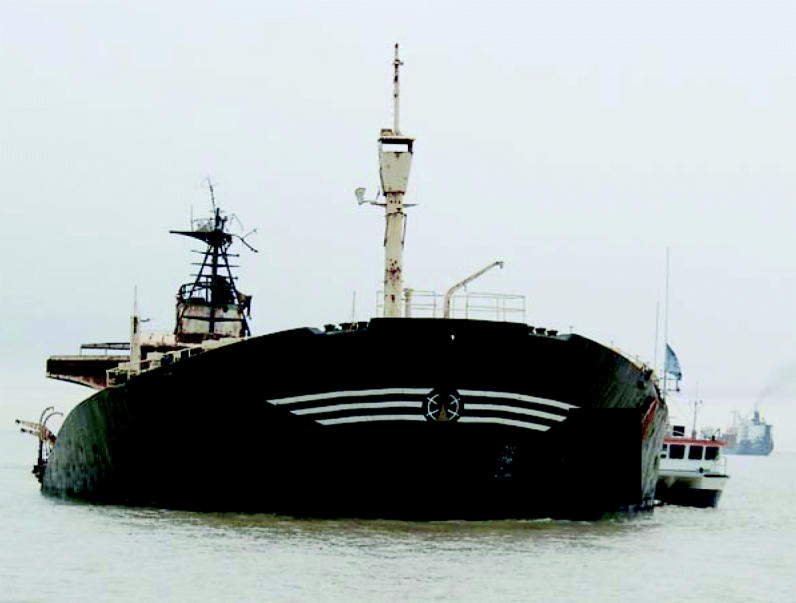

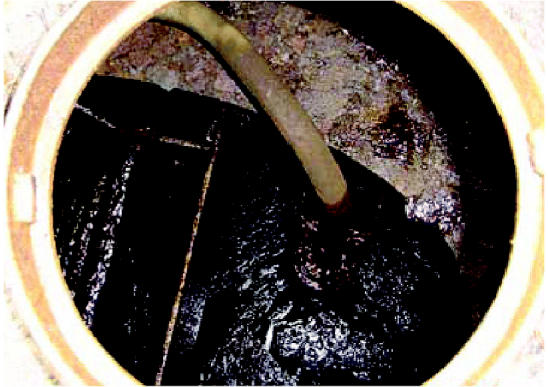
**Sea assault.** The Persian Gulf is being polluted by numerous toxic shipwrecks, such as this tanker containing 5,000 metric tons of heavy crude oil as well as three live artillery rounds that helped sink it.

